# The RNA Chaperone Hfq and Small Non-Coding RNAs Modulate the Biofilm Formation of the Fish Pathogen *Yersinia ruckeri*

**DOI:** 10.3390/ijms26104733

**Published:** 2025-05-15

**Authors:** María J. Barros, Lillian G. Acuña, Felipe Hernández-Vera, Pía Vásquez-Arriagada, Diego Peñaloza, Ana Moya-Beltrán, Fausto Cabezas-Mera, Francisco Parra, Fernando Gil, Juan A. Fuentes, Iván L. Calderón

**Affiliations:** 1 Laboratorio de RNAs Bacterianos, Centro de Investigación de Resiliencia a Pandemias, Universidad Andres Bello, Santiago 8370186, Chile; mbarrosgamonal@gmail.com (M.J.B.); hernandez.fvera@gmail.com (F.H.-V.); piacatalina.vasquez@gmail.com (P.V.-A.); diego.ignacio.mp@gmail.com (D.P.); 2 Laboratorio de RNAs Bacterianos, Departamento de Ciencias Biológicas, Universidad Andres Bello, Santiago 8370186, Chile; lillian.acuna@unab.cl; 3 Doctorado en Biotecnología, Facultad de Ciencias de la Vida, Universidad Andres Bello, Santiago 8370186, Chile; f.parralathrop@gmail.com; 4 Departamento de Informática y Computación, Facultad de Ingeniería, Universidad Tecnológica Metropolitana, Santiago 7800002, Chile; amoya@utem.cl; 5 Programa de Doctorado en Informática Aplicada a Salud y Medio Ambiente, Escuela de Postgrado, Universidad Tecnológica Metropolitana, Santiago 8330300, Chile; fcabezasme@utem.cl; 6 Laboratorio de Genética y Patogénesis Bacteriana, Centro de Investigación de Resiliencia a Pandemias, Facultad de Ciencias de la Vida, Universidad Andres Bello, Santiago 8370186, Chile; 7 Microbiota-Host Interactions & Clostridia Research Group, Center for Biomedical Research and Innovation (CIIB), Universidad de los Andes, Santiago 7620001, Chile; frgil@uandes.cl; 8 School of Medicine, Faculty of Medicine, Universidad de los Andes, Santiago 7620001, Chile

**Keywords:** Hfq, sRNA, ArcZ, RprA, RybB, biofilm, c-di-GMP, *Yersinia ruckeri*

## Abstract

The fish pathogen *Yersinia ruckeri* forms biofilms on abiotic surfaces, contributing to recurrent infections in aquaculture. Increasing evidence suggests that the RNA chaperone Hfq and small non-coding RNAs (sRNAs) are key regulators of bacterial biofilm formation. However, the regulatory mechanisms mediated by these factors remain largely unexplored in *Y. ruckeri*. In this study, we investigated the roles of Hfq and the Hfq-dependent sRNAs RprA, ArcZ, and RybB in the biofilm formation of *Y. ruckeri*. We first characterized the sRNAome of biofilm-forming cells, identifying the conserved RprA, ArcZ, and RybB, among the upregulated sRNAs. We then evaluated motility, biofilm formation, and architecture in strains lacking either *hfq* (Δ*hfq*) or these sRNAs (ΔsRNA). Our results reveal that both Δ*hfq* and ΔsRNA strains exhibit significant alterations in biofilm and motility phenotypes, including changes in bacterial morphology and extracellular matrix. Furthermore, expression analyses indicate that these sRNAs modulate the transcription of key regulatory factors, flagellar and phosphodiesterase genes, ultimately influencing intracellular cyclic di-GMP levels, a key second messenger in biofilm formation. Together, our findings demonstrate that Hfq and its associated sRNAs play critical regulatory roles in *Y. ruckeri* biofilm formation by controlling the expression of genes involved in motility, bacterial envelope proteins, and c-di-GMP metabolism.

## 1. Introduction

Bacterial biofilm formation is a fundamental survival strategy that enables microorganisms to colonize both biotic and abiotic surfaces by assembling into highly organized communities embedded within a self-produced extracellular matrix [1]. This matrix, primarily composed of polysaccharides, proteins, nucleic acids, and lipids, provides protection against antimicrobial agents and environmental stressors, thereby facilitating bacterial persistence in diverse settings ranging from natural ecosystems to industrial and clinical environments [2]. In addition to this physical protection by the extracellular matrix, bacteria inside biofilms exhibit spatial differentiation, which involves different metabolic and physiological states. This differentiation allows for efficient nutrient utilization and waste management, further enhancing resistance to antimicrobial agents and environmental stressors. For instance, some bacteria can enter in a viable but non proliferative state, by reducing metabolic activity and conserving energy. This persistence state allows bacteria to be less sensitive to some antimicrobials agents because they are not undergoing cellular processes that antibiotics can affect [3].

In aquaculture, biofilm formation is a key factor in the emergence of infectious disease outbreaks, elevated antimicrobial resistance, and persistent bacterial colonization [2,4]. These challenges often lead to the overuse of antibiotics, which can detrimentally affect fish production, compromise ecosystem integrity, and pose risks to human health. Various bacterial genera, including *Vibrio*, *Aeromonas*, *Flavobacterium*, *Streptococcus*, *Edwardsiella*, *Piscirickettsia*, and *Yersinia*, have been identified as biofilm-forming pathogens in these environments. Notably, *Yersinia ruckeri*, the causative agent of enteric redmouth disease (yersiniosis) that primarily affects salmonids in both freshwater and marine production stages, demonstrates remarkable adaptability [5]. In aquaculture systems, *Y. ruckeri* is exposed to fluctuating environmental conditions such as variations in temperature, salinity, oxygen levels, and antibiotic presence. Despite these challenges, it can survive and retain its infectivity largely due to its ability to form robust biofilms [6]. This capacity allows the pathogen to persist in aquatic sediments for months in a dormant state while maintaining its virulence [7] and to adhere strongly to common aquaculture substrates like fibrous silicate glass (fibreglass), polystyrene, polyvinyl chloride (PVC), and wood, forming stable biofilms within as little as 24 h [3,6,8].

In response to varying environmental conditions, biofilm formation is a dynamic and complex process typically encompassing four key stages: initial attachment, microcolony development, biofilm maturation, and dispersion. In *Yersinia* spp., attachment is a reversible process mediated by surface structures such as flagella and type IV pili [9]. As microcolonies develop and the biofilm matures, cells undergo three-dimensional growth while synthesizing and secreting an extracellular matrix primarily composed of water, exopolysaccharides, polypeptides, and DNA. The dispersion phase releases planktonic cells, enabling the colonization of new niches or infection of hosts [10].

Within the *Enterobacteriaceae*, the shift from a motile to a sessile lifestyle is tightly modulated by regulatory networks that integrate transcriptional and post-transcriptional controls with secondary messengers [11]. Notably, cyclic di-GMP (c-di-GMP) serves as a critical regulator; high intracellular levels promote biofilm formation by inducing exopolysaccharide synthesis, auto-aggregation, and adhesion, whereas lower levels favor motility [12]. The balance of c-di-GMP is maintained by the coordinated activities of diguanylate cyclases and phosphodiesterases, and its concentrations directly influence the production of key matrix components such as poly-N-acetyl-D-glucosamine (PNAG) in *Yersinia* spp. [13,14].

In orchestrating this complex regulatory network, increasing evidence highlights the critical role of the RNA chaperone Hfq and small non-coding RNAs (sRNAs) in modulating gene expression during the transition between planktonic and sessile lifestyles and vice-versa [15,16,17,18]. Hfq and its associated sRNAs regulate gene expression linked to motility, bacterial adhesion, and biofilm maturation; however, their regulatory impact can be strain-specific [16]. This variability underscores that findings obtained in one strain cannot be freely extrapolated to others without direct experimental validation. In this sense, the regulatory mechanisms governing biofilm formation in *Y. ruckeri* remain largely unexplored. Although quorum sensing and adhesins such as YrInv and YrIlm have been implicated in promoting biofilm formation on various abiotic substrates [6,18], the contribution of Hfq and sRNAs in this context has not been reported.

To address this gap, we characterized the sRNAome of *Y. ruckeri* biofilms and investigated the roles of Hfq and the Hfq-dependent sRNAs ArcZ, RprA, and RybB identified in our transcriptomic analysis and reported as gene expression regulators of flagellar and/or cell envelope proteins [19,20,21]. We evaluated biofilm formation, motility, architecture, and intracellular cyclic di-GMP levels using mutant strains lacking *hfq* (Δ*hfq*) and sRNAs (ΔsRNA). Based on the marked phenotypic alterations observed in Δ*rybB* and Δ*arcZ* strains, we further analyzed the expression of putative biofilm-related targets. Collectively, our findings provide compelling evidence that Hfq and sRNAs are integral components of the regulatory network modulating biofilm formation in *Y. ruckeri*.

## 2. Results

### 2.1. Determination of the sRNAome of Y. ruckeri That Forms Biofilms on an Abiotic Surface

To elucidate the regulatory mechanisms underlying *Y. ruckeri* biofilm formation, we performed a comprehensive analysis of the sRNA transcriptome in cells forming biofilms on an abiotic surface. Total RNA enriched for the small RNA fraction was extracted from wild-type cultures grown on silicate supports at 0, 4, 6, 12, and 24 h. This strategy enabled us to capture the dynamic expression profiles of sRNAs during biofilm development, thereby identifying key regulatory elements that may influence adhesion, maturation, and overall biofilm architecture.

**Figure 1:**
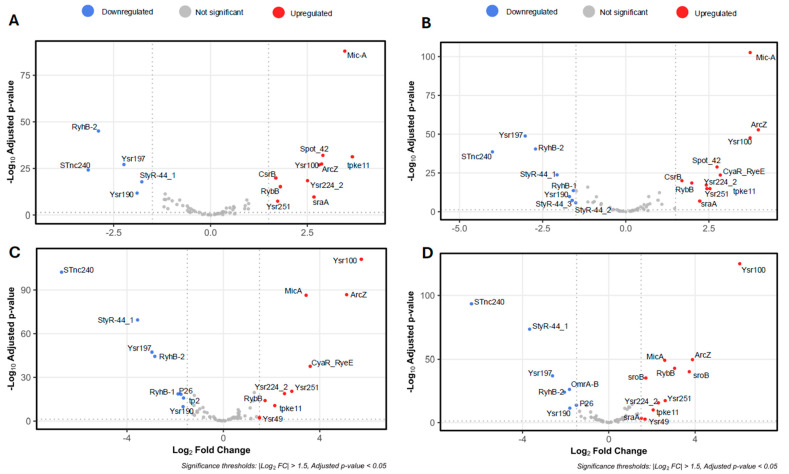
Volcano plots depicting differentially expressed sRNAs in *Y. ruckeri* biofilms. Volcano plots illustrate the differential expression of sRNAs between planktonic cells (0 h) and biofilm samples collected at (**A**) 4 h, (**B**) 6 h, (**C**) 12 h, and (**D**) 24 h. Upregulated sRNAs are shown in red, downregulated in blue, and non-significant sRNAs in gray. Functional annotations and gene identifiers are listed in Table S1. Volcano plots were generated using the R package v.4.4.1 “EnhancedVolcano” v.1.26.0.

**Figure 2:**
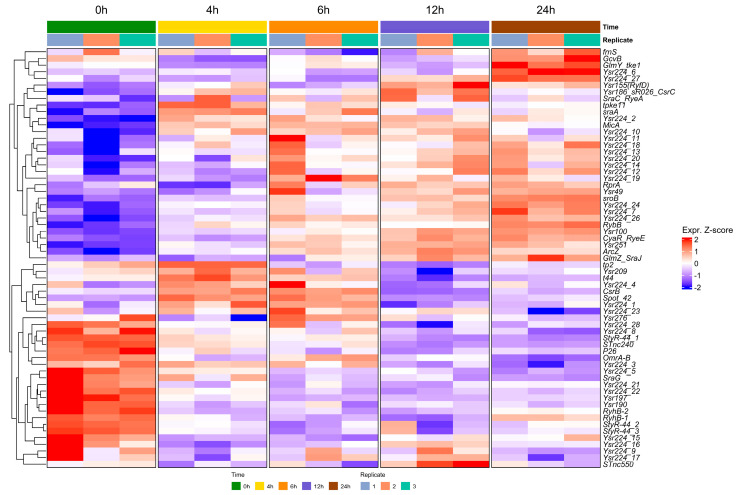
Temporal expression dynamics of top-ranked sRNAs during biofilm development by *Y. ruckeri*. The heatmap displays expression profiles of the 70 most significantly differentially expressed sRNAs across five biofilm time points: 0 h, 4 h, 6 h, 12 h, and 24 h. Each column corresponds to one of three biological replicates per time point, while rows represent individual sRNAs.

To explore the transcriptional dynamics of sRNA expression during biofilm formation, volcano plots and heatmaps were used to visualize differentially expressed sRNAs identified through DESeq2 analysis based on normalized counts. Visualizations were generated using the R packages via ggplot2, Heatmapper, and VolcaNoseR8 [22,23]. Figure 1 provides the differential expression profiles across four biofilm development times (4, 6, 12, and 24 h), each compared to the planktonic condition (0 h). Two dominant regulatory patterns emerged: a subset of sRNAs showed progressive upregulation as the biofilm matured, while others were significantly downregulated during the transition from planktonic to sessile growth. In the heatmap, we observed the expression dynamics of the most representative RNAs during biofilm development, among which we can identify conserved sRNAs previously reported in the biofilm process in enterobacteria (Figure 2). In addition, *Yersinia*-specific sRNAs (Yrs) appeared, which have not previously been linked or characterized under biofilm conditions. The sRNAs identified in the trancriptomic analysis were named based on the sequence identity (>80%) with sRNAs categorized in the Rfam database [24] (Table S1). Interestingly, transcripts corresponding to sRNAs with more than one copy are observed, as in the case of Ysr224, whose multiple copies show percentages of identity ranging from 80 to 91%, but with different expression profiles during biofilm formation (Figure 2).

At 0 h, corresponding to planktonic phase cells just prior to incubation and biofilm formation, sRNAs such as MicA, SraA, Spot42, and CsrB exhibited low expression but in the transition to early times of biofilm formation (2 h) were significantly induced. On the other hand, sRNAs such as RyhB-1, RyhB-2, OmrA/B, StyR-44, Ysr190, and Ysr197 were expressed at higher levels in planktonic cells (0 h), but were drastically downregulated in the transisiton to 2 h of biofilm formation. By 4 h, many sRNAs showed a progressive increase in expression, with MicA and SraA standing out as among the most upregulated, indicative of early regulatory roles at biofilm initiation. At 6 h, the expression profile became heterogeneous: some sRNAs, including CsrB and Spot42, remained consistently upregulated (Z-score > 1), whereas others, such as Tp2 sRNA, were downregulated, suggesting tha it is no longer required at this stage. In addition, others appeared that increased their expression at this stage, e.g., ArcZ, CyaR, RprA, and Ysr251. At 12 h, SroB and Ysr49 exhibited significant upregulation, plausibly linked to biofilm maturation. By 24 h, sRNAs with a more discrete expression in earlier stages showed significant upregulation at this later stage, e.g., RybB, GcvB, and GlmY (Figure 2). These dynamic expression patterns underscore a functional differentiation among sRNAs, with some probably directly involved in the formation of biofilm, while others act as regulators of metabolic homeostasis.

### 2.2. Hfq Is Required for the Stability of sRNAs Expressed During the Biofilm Formation of Y. ruckeri

To further investigate the functions of some of the sRNAs identified in the transcriptomic analysis, we selected ArcZ, RprA, and RybB, which showed different induction profiles at all times of biofilm formation. In addition, these sRNAs have been reported as regulators of gene expression for flagellar and/or cell envelope proteins in *Enterobacteriaceae*, as well as of biofilm formation [19,20,21].

**Figure 3:**
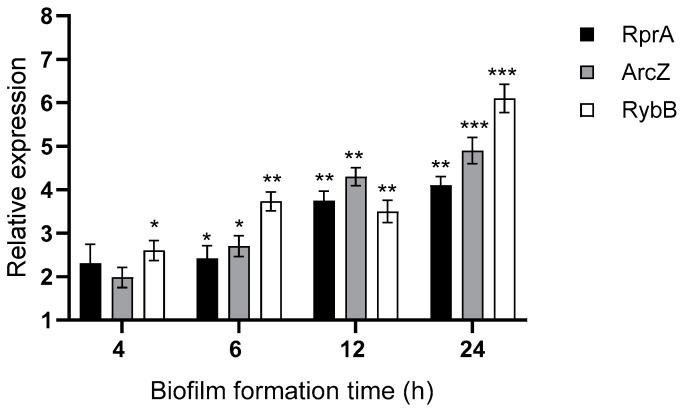
Relative expression of ArcZ, RprA, and RybB sRNAs in *Y. ruckeri* that forms biofilms. Wild-type strain was grown without shaking at 22 °C on silicate covers for the indicated times. Adherent cells were recovered by low-frequency sonication and subjected to total RNA extraction. Gene expression was quantified by RT-qPCR and is shown relative to the time 0 h of biofilm formation (initial inoculum of planktonic cells). Asterisks represent statistically significant differences with respect to the time 0 h (*** *p* < 0.001, ** *p* < 0.01, * *p* < 0.05). Data represent the means ± standard deviations (*n* = 3).

**Figure 4:**
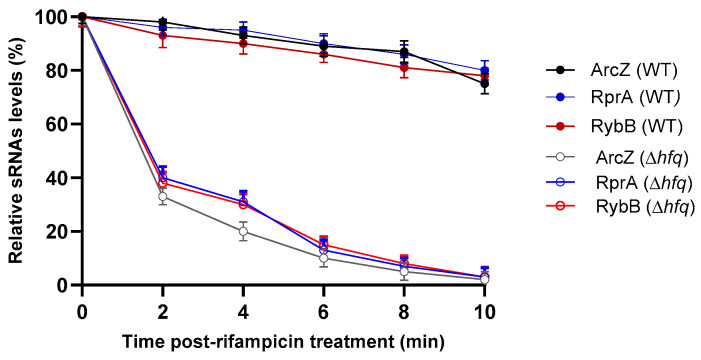
Hfq-dependent stability of *Y. ruckeri* sRNAs. sRNA stability was assessed in wild-type (WT) and ∆*hfq* strains following transcription inhibition with rifampicin. Total RNA was extracted at the designated time points and quantified using RT-qPCR. sRNA levels are expressed as a percentage relative to the initial time point (0 min). Data represent the means ± standard deviations (*n* = 3).

First, we validated the expression of these sRNAs at different times of biofilm formation by qRT-PCR, confirming the transcriptomic profiling observed above (Figure 3). Since the Hfq-dependent stability of most sRNAs is critical for effective post-transcriptional regulation by base-paring with target mRNAs, we compared the decay rates of the *arcZ*, *rprA*, and *rybB* transcripts in wild-type and Δ*hfq* strains that form biofilms. Transcription was inhibited using rifampicin, and sRNA decay was monitored over time. As shown in Figure 4, all examined sRNAs exhibited significantly shorter half-lives in the Δ*hfq* strain compared to the wild type, confirming that Hfq is essential for sustaining sRNA stability under biofilm conditions. This destabilization suggests that these specific Hfq-sRNA complexes may contribute to the regulatory networks involved in *Y. ruckeri* biofilm formation. In this context, we focused the following analyses on these three Hfq-dependent sRNAs.

### 2.3. Hfq and the sRNAs ArcZ, RprA, and RybB Differentially Modulate Biofilm Formation in Y. ruckeri

#### 2.3.1. Hfq and the sRNAs ArcZ, RprA, and RybB Are Required for the Motility Regulation of *Y. ruckeri*, a Pivotal Trait for Specific Stages of Biofilm Formation

**Figure 5:**
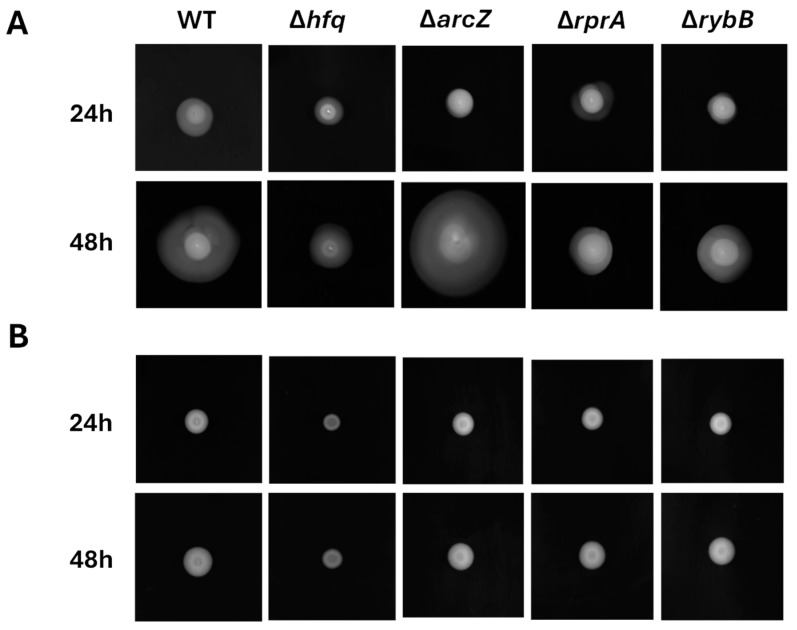
Motility assays of *Y. ruckeri* strains. Late-log phase cultures (1 µL) of wild-type (WT), Δ*hfq*, and ΔsRNA strains were spotted onto TSA agar plates containing 0.3% agar for swimming assays (**A**) and 0.5% agar for swarming assays (**B**). Motility was evaluated after 24 and 48 h of incubation. Representative results from five independent experiments are shown.

Motility is crucial during the early stages of biofilm formation, as it enables bacteria to locate and adhere to abiotic surfaces. To assess whether Hfq and its associated sRNAs (ArcZ, RprA, and RybB) influence this trait, we conducted swimming and swarming assays on 0.3% and 0.5% (*w*/*v*) TSA agar, respectively, incubating the plates for 24 and 48 h. The Δ*hfq* strain exhibited a marked reduction in swimming at 48 h (Figure 5A) and reduced swarming at both 24 and 48 h (Figure 5B). By contrast, Δ*arcZ* demonstrated enhanced swimming at 48 h, whereas Δ*rprA* and Δ*rybB* mutants showed significantly decreased swimming at both time points (Figure 5A). Swarming remained unchanged in Δ*arcZ*, Δ*rprA*, and Δ*rybB* strains compared to the wild type (Figure 5B). These findings highlight the importance of Hfq and these sRNAs in modulating motility, a key factor not only for both initial surface attachment, but also for biofilm dispersal. The diameter of motility halos and statistical significances are shown in Supplementary Figure S1.

#### 2.3.2. Hfq and the sRNAs ArcZ, RprA, and RybB Participate at Different Stages of Biofilm Formation, Modulating the Attachment and/or the Architecture of *Y. ruckeri* Conglomerates

**Figure 6:**
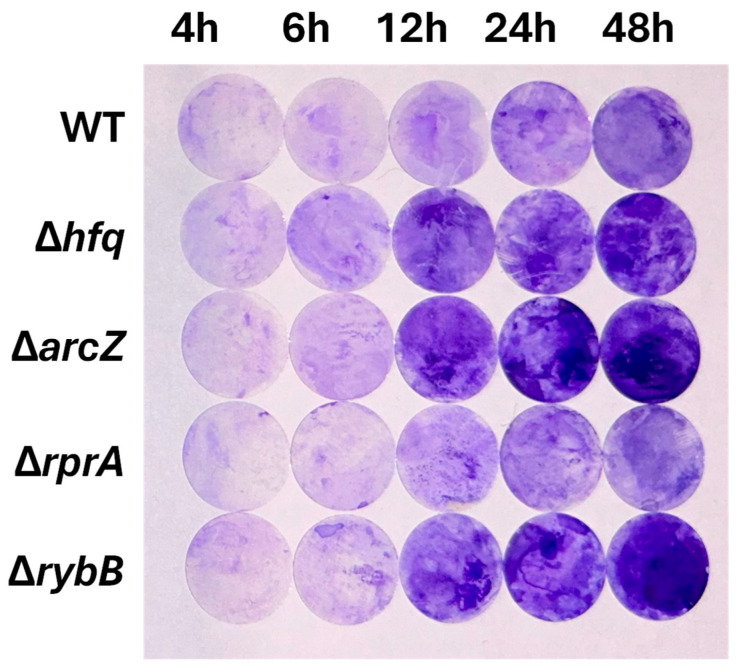
Crystal violet biofilm assays for *Y. ruckeri* strains. Wild-type (WT), Δ*hfq*, and ΔsRNA strains were grown on silicate covers without shaking at 22 °C for the indicated durations (4, 6, 12, 24, and 48 h). The covers were then stained with 1% (*v*/*v*) crystal violet to visualize biofilm formation. Representative images from each time point are shown (*n* = 5).

To investigate the impact of Hfq and the ArcZ, RprA and RybB sRNAs on biofilm formation in *Y. ruckeri*, we performed a Crystal Violet (CV) staining assay using wild-type, Δ*hfq*, and ΔsRNA strains cultured on silicate slides for 4, 6, 12, 24, and 48 h. Our results indicate that, during the early stages of biofilm development (4 and 6 h), the absence of Hfq does not significantly alter biofilm formation. Additionally, at later time points, where we expect more developed biofilms (12, 24, and 48 h), the Δ*hfq* strain exhibited a significant increase in biofilm formation (Figure 6), suggesting a modulatory role for Hfq and its dependent sRNAs. Among these Hfq-dependent sRNAs are ArcZ, RprA, and RybB, which precisely showed higher expression at late time points (Figure 3). The deletion of RprA sRNA did not show alteration in biofilm formation compared to the wild type, whereas the absence of ArcZ and RybB resulted in increased biofilm formation from 12 h onward (Figure 6). These findings indicate that the regulatory role of Hfq, along with the sRNAs ArcZ and RybB, impacts in the final output of *Y. ruckeri* biofilm formation. The absorbance at 570 nm and statistical significances are shown in Supplementary Figure S2. 

To evaluate the impact of Hfq and its dependent sRNAs on biofilm architecture, we performed qualitative analyses by confocal microscopy. All strains were transformed with a plasmid constitutively expressing green fluorescent protein (pDiGc plasmid, [25]) and allowed to form biofilms on silicate covers for 4, 6, 12, 24, and 48 h.

**Figure 7:**
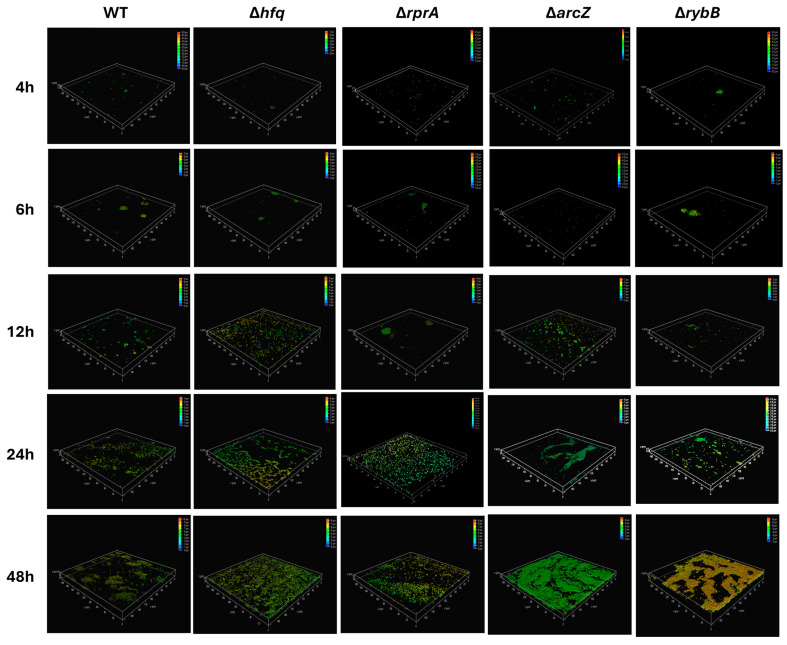
Confocal microscopy analysis of *Y. ruckeri* biofilms. Wild-type (WT) and mutant strains (Δ*hfq*, Δ*rprA*, Δ*arcZ*, and Δ*rybB*) were grown on silicate covers without shaking at 22 °C for the indicated times (4, 6, 12, 24, and 48 h). After incubation, covers were washed and fixed with 4% paraformaldehyde. Samples were visualized on a Leica TCS SP8 confocal microscope at 6000× magnification, and images were reconstructed using LAS X software 3.1.5. The color scale (heatmap) in each panel represents the depth (Z-axis) of the biofilm in micrometers (µm). Representative images from each time point are shown (*n* = 3).

Figure 7 provides a detailed three-dimensional view of biofilm architecture for wild-type and mutant *Y. ruckeri* strains as visualized by confocal microscopy. The reconstructed images, which include a heatmap indicating biofilm depth in micrometers, reveal several key observations. At early time points (4 and 6 h), biofilms appear relatively similar among the strains, although subtle differences in microcolony formation can be noted (compare the wild type with Δ*rprA* and Δ*rybB*). As biofilm development proceeds (12, 24, and 48 h), pronounced architectural differences emerge. Notably, the Δ*hfq* strain forms a biofilm with increased vertical growth and density, reaching heights of approximately 6 μm by 24 h, significantly exceeding the wild-type biofilm, which remains below 3 μm at these stages (Figure 7). Among the ΔsRNA mutants, differential patterns are also evident. The Δ*arcZ* and Δ*rybB* strains exhibit alterations in biofilm density and height; for example, Δ*rybB* biofilms tend to be taller, whereas Δ*arcZ* biofilms show an increased accumulation of biomass. In contrast, the Δ*rprA* mutant demonstrates a more dispersed architecture with fewer and less densely packed microcolonies during the early phases, although these differences become more apparent as the biofilm matures (Figure 7). Overall, the confocal microscopy analysis confirm that the regulatory roles of Hfq and these sRNAs in biofilm development ultimately impacts on the structural characteristics of the biofilm formed.

**Figure 8:**
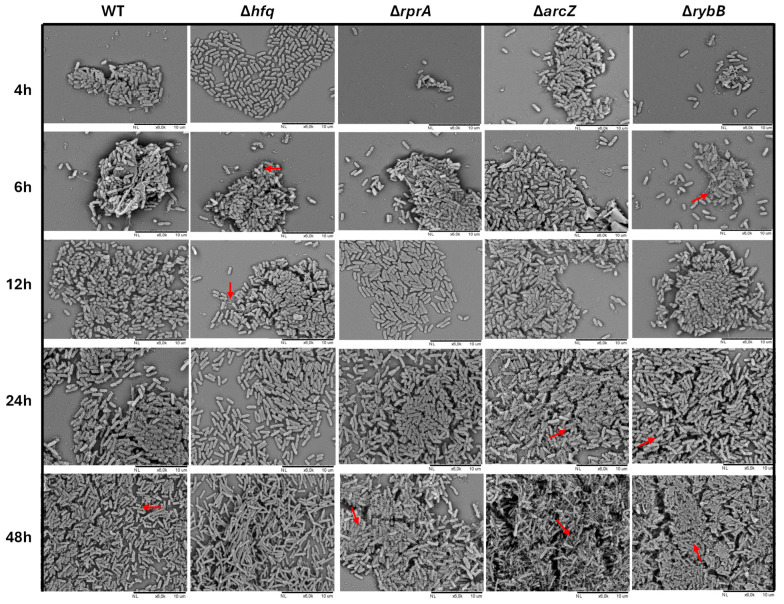
Scanning electron microscopy analysis of *Y. ruckeri* biofilms. Wild-type (WT), Δhfq, and ΔsRNA (Δ*rprA*, Δ*arcZ*, and Δ*rybB*) strains were grown on silicate covers at 22 °C for the indicated times without shaking. After incubation, covers were washed and fixed with 2.5% glutaraldehyde, followed by critical point drying and gold coating. Samples were then imaged at 6.0 K magnification using a Hitachi TM3000 microscope (Hitachi High-Tech, Tokyo, Japan), providing a detailed view of the biofilm architecture under each condition. Red arrows indicating extracellular components and/or appendages. Representative images from each time point are shown (*n* = 3).

To assess in more detail the architecture of the biofilm matrix, scanning electron microscopy (SEM) was performed on biofilms formed by all strains (Figure 8). At early stages (4 h), the Δ*hfq* strain primarily formed monolayers on the support, with bacteria connected by cellular appendages, in contrast to the wild type, which already exhibited three-dimensional microcolonies enriched with extracellular components. As biofilm development progressed (24 and 48 h), these differences became more pronounced. The Δ*hfq* strain demonstrated denser biomass compared to the wild type; however, it showed fewer extracellular appendages and more elongated cell morphology, especially at 48 h. Among the ΔsRNA mutants, significant differences emerged at later time points, with the Δ*arcZ* and Δ*rybB* strains exhibiting increased surface coverage of cells accompanied by extracellular components (Figure 8). Collectively, these SEM observations, together with the results from motility and Crystal Violet assays, indicate that Hfq and its associated sRNAs (ArcZ, RybB, and RprA) play distinct, non-redundant roles in modulating biofilm formation in *Y. ruckeri*. Notably, the absence of RprA appears to have more impact on early stages of biofilm formation, while deficiencies in ArcZ and RybB have a more substantial impact on the later stages of biofilm maturation, which is consistent with their dynamic expression profiles (Figure 3).

#### 2.3.3. Hfq and the sRNAs ArcZ and RybB Modulate the Production of Extracellular Polysaccharides in *Y. ruckeri* Biofilms

Since poly-N-acetyl-D-glucosamine (PNAG) is one of the principal polysaccharides in *Yersinia* spp. and in many bacteria [26], we examined its production in the biofilms of *Y. ruckeri* Δ*hfq* and ΔsRNA strains. All strains were pre-transformed with the pDiGc plasmid, and PNAG was detected using Alexa Fluor 647-conjugated wheat germ agglutinin (WGA-AF647, red fluorescence), which preferentially binds N-acetylglucosamine and sialic acid residues [27,28].

**Figure 9:**
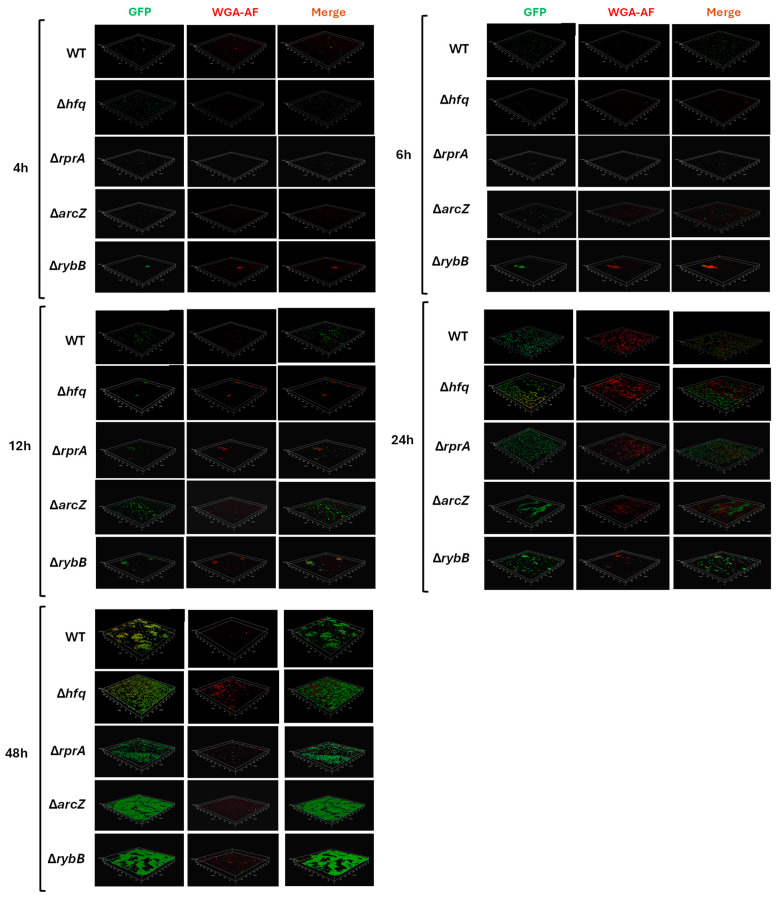
Confocal microscopy analysis of poly-N-acetyl-D-glucosamine (PNAG) production in *Y. ruckeri* biofilms. Wild-type (WT), Δ*hfq*, and ΔsRNA strains were grown on silicate covers without shaking at 22 °C for the indicated times. After incubation, covers were washed, stained with Alexa Fluor 647-conjugated wheat germ agglutinin (WGA), and fixed in 4% (*v*/*v*) paraformaldehyde. In each panel, the left column (GFP) shows green-labeled bacteria, the middle column (WGA-AF) indicates PNAG abundance, and the right column (merge) shows the overlay of both channels. Samples were imaged using a Leica TCS SP8 confocal microscope at 6000× magnification, and images were reconstructed with the LAS X software. Representative images from each time point are shown (*n* = 3).

At 4 h of biofilm formation, PNAG was present in all strains with no noticeable differences (Figure 9. However, at later time points (24 and 48 h), the Δ*hfq* strain exhibited a significantly higher density of PNAG compared to the wild type (Figure 9). In contrast, the deletion of the RprA sRNA did not showed differences in PNAG levels at any examined time point (Figure 9). The Δ*arcZ* strain displayed no significant differences at early stages (4 and 6 h); yet, by 12 h, an increased PNAG density relative to the wild type was evident, a difference that became more pronounced at 24 h (Figure 9). Interestingly, at 48 h, the Δ*arcZ* strain showed a marked decrease in the PNAG signal, suggesting a subsequent reduction in polysaccharide production. For the Δ*rybB* strain, initial increases in cell density correlated with elevated PNAG levels; however, similar to Δ*arcZ*, a decrease in red fluorescence was observed at 48 h, indicating a lower PNAG density than in the wild type.

**Figure 10:**
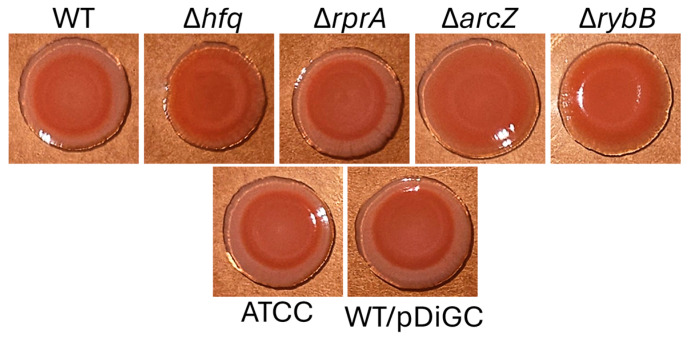
Macrocolony morphology of *Y. ruckeri* strains on Congo red agar. Three microliters of overnight cultures grown in TSB were spotted onto 0.08% Congo red agar plates and incubated at 22 °C for 72 h. The image shown is representative of three independent experiments. “ATCC” refers to the wild-type control strain ATCC 29473, while “WT/pDiGc” corresponds to the CD2 strain transformed with the pDiGc plasmid. Representative images from three independent assays.

In parallel, we employed Congo red staining to further assess biofilm matrix production (Figure 10). The macrocolony morphology in Congo red agar revealed that the Δ*hfq*, Δ*arcZ*, and Δ*rybB* strains exhibited enhanced Congo red binding, consistent with an overproduction of extracellular polysaccharides. Variations in the thickness, color intensity (ranging from pink to red), and ring patterns of the macrocolonies underscore distinct differences in biofilm matrix structure among the strains.

Collectively, these results suggest that Hfq and the sRNAs ArcZ and RybB play important roles in modulating the metabolism and/or export of PNAG and potentially other biofilm matrix components in *Y. ruckeri*.

### 2.4. Hfq and the sRNA ArcZ Modulate the Intracellular Levels of c-di-GMP in Y. ruckeri

**Figure 11:**
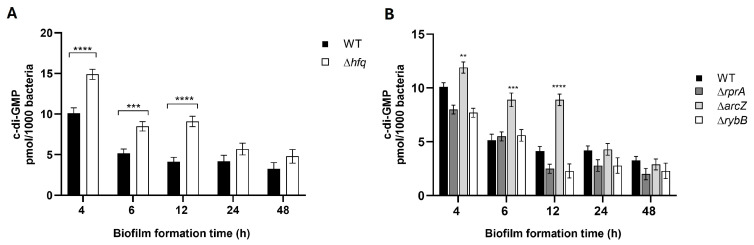
Intracellular c-di-GMP levels in *Y. ruckeri* biofilms. Wild-type (WT), Δ*hfq*, and ΔsRNA strains were grown without shaking at 22 °C on silicate covers for the indicated times. Adherent cells were recovered by low-frequency sonication, resuspended in phosphate buffer, and subjected to c-di-GMP quantification by ELISA (469/501 nm). Results represent three independent replicates. (**A**) Comparison between WT and Δ*hfq*. (**B**) Comparison between WT and Δ*arcZ*, Δ*rprA*, and Δ*rybB*. Asterisks represent statistically significant differences with respect to the WT (**** *p* < 0.0001; *** *p* < 0.001, ** *p* < 0.01). Data represent the means ± standard deviations (*n* = 3).

Given that the mutant strains exhibited altered biofilm density and differential polysaccharide production, we next evaluated the intracellular levels of c-di-GMP, a key second messenger implicated in biofilm regulation in other *Enterobacteriaceae* [12]. In this assay, biofilms of wild-type, Δ*hfq*, and ΔsRNAs strains were formed under identical conditions, and cells were recovered to quantify c-di-GMP levels by ELISA. As shown in Figure 11, the Δ*hfq* strain displayed significantly elevated c-di-GMP levels from 4 to 12 h of biofilm formation compared to the wild type (Figure 11A). Similarly, the Δ*arcZ* strain exhibited a pronounced increase in c-di-GMP, particularly at 12 h (Figure 11B). These findings suggest that both Hfq and ArcZ are involved in maintaining c-di-GMP homeostasis, likely by regulating the expression of genes associated with c-di-GMP metabolism and thereby influencing the biofilm phenotype of *Y. ruckeri*.

### 2.5. ArcZ and RybB Regulate the Expression of Biofilm-Related Genes in Y. ruckeri

Given the pronounced and pleiotropic effects observed following the deletion of *arcZ* and *rybB*, including altered motility, denser biofilms, and, in the case of *arcZ*, elevated intracellular c-di-GMP levels, we sought to identify potential targets for these sRNAs that could underlie these phenotypes. Target prediction was performed using the CopraRNA algorithm [29,30,31], which evaluates both the accessibility of interaction sites and the conservation of putative targets. For this analysis, we examined regions spanning from 200 nucleotides upstream to 100 nucleotides downstream of the annotated start codon, as most reported sRNA–mRNA interactions occur within this window.

**Figure 12:**
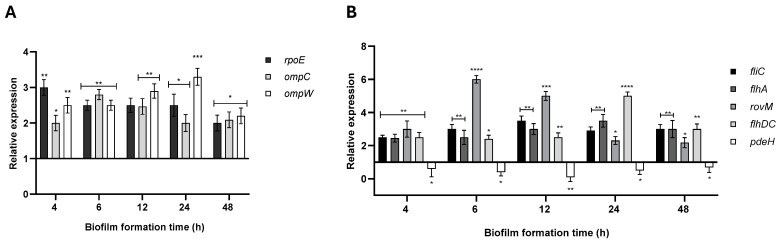
Relative expression of putative RybB and ArcZ target genes in *Y. ruckeri*. Wild-type (WT), Δ*rybB*, and Δ*arcZ* strains were grown without shaking at 22 °C on silicate covers for the indicated times. Adherent cells were recovered by low-frequency sonication and subjected to total RNA extraction. Gene expression was quantified by RT-qPCR and is shown relative to WT. (**A**) *rpoE*, *ompC*, and *ompW* in Δ*rybB*. (**B**) *fliC*, *flhA*, *rovM*, *flhDC*, and *pdeH* in Δ*arcZ*. Asterisks represent statistically significant differences with respect to the WT (**** *p* < 0.0001; *** *p* < 0.001; ** *p* < 0.01; * *p* < 0.05). Data represent the means ± standard deviations (*n* = 3).

For RybB, the predicted targets primarily included mRNAs encoding outer membrane proteins (OMPs), such as OmpC (CopraRNA *p*-value = 0.095) and OmpW (CopraRNA *p*-value = 0.007), as well as the envelope stress transcriptional regulator RpoE (CopraRNA *p*-value = 0.003). The expression analysis in biofilms formed by the Δ*rybB* strain, relative to the wild type under identical conditions, revealed a significant upregulation of all predicted targets (Figure 12A), supporting a repressive role for RybB on these genes.

Similarly, for ArcZ, the predictive algorithm identified flagellar mRNAs *fliC* (CopraRNA *p*-value = 0.0004) and *flhA* (CopraRNA *p*-value = 0.012), and the mRNA of the transcriptional regulator RovM (CopraRNA *p*-value = 0.004). Notably, RovM regulates the flagellar master operon *flhDC* in *Y. pseudotuberculosis* and modulates virulence genes [32], while *flhDC*, in turn, has been implicated in controlling the global phosphodiesterase PdeH in *E. coli* [33]. To explore this potential regulatory network in *Y. ruckeri*, we analyzed the expression of *rovM*, *flhDC*, and *pdeH* in biofilms formed by the Δ*arcZ* strain. Our data showed a significant upregulation of *rovM* and *flhDC* at 12, 24, and 48 h (Figure 12B), indicating that ArcZ normally represses their expression. Conversely, *pdeH* expression was downregulated in the absence of ArcZ, suggesting that ArcZ may positively regulate PdeH. Additionally, the increased expression of *fliC* and *flhA* in the Δ*arcZ* strain further supports a repressive role for ArcZ on these targets.

Together, these expression profiles align with the phenotypic alterations observed in the Δ*arcZ* and Δ*rybB* strains, highlighting the key roles of these sRNAs in modulating the regulatory networks that govern biofilm formation in *Y. ruckeri*.

## 3. Discussion

*Yersinia* ruckeri forms biofilms on abiotic surfaces commonly found in fish farms, leading to recurrent infections. This phenomenon not only adversely affects aquaculture but also has significant ecological consequences, as biofilm-associated bacteria necessitate the extensive use of antimicrobial agents. Developing alternative antimicrobial strategies for these pathogens, e.g., those focused in inhibiting molecular systems and factors that mediate biofilm formation [34], requires a thorough understanding of the molecular mechanisms underlying biofilm formation. Therefore, this study aimed to elucidate the roles of the RNA chaperone Hfq and small non-coding RNAs (sRNAs) in the biofilm formation of *Y. ruckeri*. Although Hfq and sRNAs are well-known mediators of bacterial responses to stress and environmental changes, their involvement in the biofilm formation of this salmonid pathogen has not been investigated.

### 3.1. sRNAome of Yersinia ruckeri That Forms Biofilms Revealed Newly and Conserved sRNAs

In this study, we employed high-throughput RNA sequencing to profile small regulatory RNAs (sRNAs) expressed under biofilm conditions in *Y. ruckeri*. To our knowledge, this represents the first comprehensive transcriptomic screening of sRNAs in this species, resulting in the identification of numerous sRNAs with distinct expression patterns during biofilm development. The analysis revealed two major groups: sRNAs that are downregulated and those that are upregulated as biofilm formation progresses. These dynamic patterns suggest that the identified sRNAs play critical, non-redundant roles in both the transition from planktonic to sessile states and in the maturation of biofilms, likely through targeting different sets of mRNAs.

The sRNA repertoire includes both conserved elements, such as ArcZ, RprA, RybB, MicA, and GcvB, which have been previously implicated in biofilm regulation in *Enterobacteriaceae*, and novel sRNAs that have not been associated with biofilm formation before. Additionally, several sRNAs previously characterized in *Y. pestis* and *Y. pseudotuberculosis* as virulence factors under infective conditions were detected [35,36,37], underscoring their potential contribution to pathogenicity. These findings suggest that biofilm-related sRNAs may facilitate immune evasion, reduce antimicrobial efficacy, and promote persistent infections [38,39].

A particularly noteworthy outcome of our analysis is the identification of sRNAs that might be unique to *Y. ruckeri*, reflecting its distinct evolutionary trajectory within the *Yersinia* genus. As this pathogen occupies a distinct basal lineage within the *Yersinia* genus, it is plausible that it has evolved specialized regulatory elements tailored to its ecological niche [40]. Further investigations are essential to elucidate the roles of these novel sRNAs in biofilm formation and pathogenicity, potentially revealing new targets for therapeutic intervention.

### 3.2. Differential Correlation Among the Motility Phenotypes and Biofilm Outputs of Y. ruckeri Strains Indicates That They Operate at Different Nodes of the Regulatory Network

Biofilm formation is intrinsically linked to cell motility, which facilitates the transition from a planktonic to a sessile lifestyle [41]. Under our experimental conditions, the absence of Hfq resulted in reduced swimming motility (Figure 5), a phenotype consistent with observations in other aquatic pathogens such as *Vibrio harveyi* [42], although contrasting with reports in *Yersinia pseudotuberculosis* where Hfq deletion increased motility [43]. Interestingly, despite the diminished motility, the Δ*hfq* strain formed biofilms with greater surface coverage at later time points, albeit with altered matrix characteristics. Regarding Hfq-dependent sRNAs, differential impacts on motility were further revealed. Specifically, the deletion of *arcZ*, *rybB,* and *rprA* produced distinct motility phenotypes: Δ*arcZ* exhibited increased swimming motility, suggesting that ArcZ normally acts as a repressor, whereas Δ*rprA* and Δ*rybB* displayed reduced motility, consistent with activator roles. Instead, in *E. coli*, ArcZ represses both swimming and swarming motilities, and RprA has a repressor function only on swarming motility, whereas the overexpression of RybB does not induces changes in *E. coli* motility [44]. Different regulatory functions of these sRNAs on motility were also found in the plant pathogen *Erwinia amylovora,* where ArcZ and RprA act as positive regulators of swimming motility [45,46]. These differential functions of Hfq and sRNAs on flagellar-dependent motility among bacteria could be explained by the pleiotropic role of Hfq as chaperone of different sRNAs and the ability of many sRNAs to regulate the expression of multiple targets. In addition, the diversity of sRNAs and their functions also varies among species and have been evolutionarily shaped by the environmental characteristics in which bacteria thrive [47].

Furthermore, Hfq–sRNA complexes not only directly regulate genes associated with motility and biofilm formation but also modulate key transcriptional factors, such as RpoS, FlhDC, and CsgD [11,48,49], which further integrate multiple environmental and cellular signals. The observed motility defects and corresponding biofilm phenotypes in the Δ*hfq*, Δ*arcZ*, Δ*rprA*, and Δ*rybB* strains suggest that these regulatory molecules operate at different nodes within the biofilm regulatory network. For instance, the enhanced motility in Δ*arcZ* correlates with increased biofilm formation, whereas the reduced motility in Δ*rprA* does not appear to adversely affect biofilm maturation, and the reduced motility in Δ*rybB* is associated with increased biofilm formation.

Collectively, these differential phenotypes underscore the non-redundant roles of these sRNAs and highlight the complexity of the regulatory mechanisms governing biofilm formation in *Y. ruckeri*. Such complexity is further supported by studies in *E. coli*, where the overexpression of multiple sRNAs yielded diverse and sometimes non-correlated effects on biofilm formation [44], reflecting the intricate interplay among regulatory pathways in bacterial biofilm development.

### 3.3. Hfq and sRNAs Modulate the Biofilm Formation Ability of Yersinia ruckeri by Maintaining the Balance on Gene Expression, Metabolism, and Production of Key Factors Related to Biofilm Development

The architecture and composition of a bacterial biofilm are heavily influenced by the support material and the underlying regulatory networks controlling gene expression, metabolism, and extracellular matrix production. In our study, silicate support, a material commonly used in fish farming facilities, provided a consistent substrate to correlate phenotypic observations across multiple assays.

As expected, the deletion of *hfq* led to significant alterations in biofilm formation, with the Δ*hfq* strain displaying markedly increased biofilm biomass at later time points (24 and 48 h) (Figure 4, Figure 5, Figure 6 and Figure 7). This observation is consistent with previous reports in *Y. pestis*, where the loss of Hfq resulted in enhanced biofilm formation [50]. Several factors may contribute to this phenotype, including the indirect effects of reduced motility, which favor the establishment of a sessile state, and the direct regulatory influence of Hfq on the expression of genes critical for biofilm maturation, such as those involved in c-di-GMP metabolism and exopolysaccharide synthesis [17].

Indeed, the quantification of intracellular c-di-GMP revealed that the Δ*hfq* strain exhibits elevated levels of this second messenger during the early stages of biofilm development, suggesting a dysregulation of c-di-GMP homeostasis. This imbalance likely leads to the formation of biofilms with increased biomass and an extracellular matrix enriched in PNAG compared to the wild type. Moreover, the increased red fluorescence observed in confocal micrographs of the Δ*hfq*, Δ*arcZ*, and Δ*rybB* strains (Figure 7) supports the notion of enhanced PNAG accumulation. However, caution is warranted, as the Alexa Fluor-conjugated WGA used for PNAG detection may also bind to other extracellular components, such as lipopolysaccharides [51], which are also modulated by Hfq-sRNA complexes [52]. Regarding the observed reduction in PNAG levels at 48 h in the Δ*arcZ* and Δ*rybB* strains (Figure 7), previous studies have shown that the fluorescence emission of Alexa Fluor can be quenched by proteins or amino acids in the environment, which can affect quantification [53]. Consequently, it is plausible that alterations in the overall composition of the biofilm matrix in these mutant strains, specifically changes in protein and/or amino acid content, may interfere with the fluorescence signal and lead to an apparent reduction in PNAG levels.

Scanning electron microscopy (SEM) further revealed that Δ*hfq* cells tend to exhibit an elongated morphology, suggesting a role for Hfq in regulating cell division, an observation consistent with previous findings in *E. coli* where Hfq influences the expression of key division genes (e.g., *zipA*, *ftsN*, and *ftsZ*) [54,55]. The disruption of cell division may contribute to the delayed formation of three-dimensional microcolonies at early biofilm stages [56]. However, by 48 h, the Δ*hfq* strain ultimately forms a denser biomass that, despite its increased coverage, appears to have an imbalanced extracellular matrix composition. Such an imbalance could compromise the biofilm’s protective function, as we have previously observed that Δ*hfq* cultures, despite faster replication and higher ATP levels, exhibit defects in persister cell formation upon antibiotic exposure [57].

The imbalanced c-di-GMP levels observed in the Δ*hfq* strain likely contribute to its altered biofilm phenotype. The second messenger c-di-GMP regulates not only exopolysaccharide production but also the expression of adhesins and other structural components of the extracellular matrix [58]. In *Y. pestis*, Hfq directly modulates c-di-GMP concentrations by controlling the expression of the diguanylate cyclase HmsT and the phosphodiesterase HmsP [50]. Specifically, Hfq enhances the stability of *hmsP* mRNA, leading to increased HmsP protein levels while concurrently reducing the stability of *hmsT* mRNA, thereby decreasing HmsT protein production. This dual regulatory mechanism is essential for maintaining low yet optimal levels of c-di-GMP.

Regarding the microscopic characterization of biofilms in ΔsRNA strains, the most pronounced differences emerge at later stages of biofilm formation. Both the Δ*arcZ* and Δ*rybB* strains exhibit greater surface coverage, increased biomass density, and enhanced production of extracellular matrix components and PNAG. In *E. coli* and *Salmonella enterica*, sRNAs such as RybB and RprA directly target the mRNA encoding CsgD, a key transcriptional activator of biofilm formation, thereby repressing its expression and reducing the production of extracellular matrix components like curli fibers and cellulose [59,60]. Additionally, ArcZ modulates biofilm formation in organisms such as *S. enterica* sv. Typhimurium and *E. coli* by both directly and indirectly regulating CsgD expression, as well as through the post-transcriptional control of FlhDC, the transcriptional activator of flagellar synthesis [61,62].

In *Y. ruckeri*, the increased biofilm formation observed in the absence of ArcZ and RybB likely results from the dysregulation of one or more critical genes involved in the synthesis of major extracellular matrix components. Notably, no homolog of CsgD has been identified in *Yersinia*, and PNAG is the principal polysaccharide in the biofilm matrix of this genus [13,14]. In species such as *Y. pestis* and *Y. pseudotuberculosis*, PNAG is synthesized and exported by components of the hemin storage locus, specifically the *hmsHFRS* operon, which is orthologous to the *pgaABCD* operon in *E. coli* [63,64,65]. Many *Y. ruckeri* strains, including the CD2 strain used in this study, harbor a putative biofilm adhesin polymer cluster partially associated with these operons; however, its function remains uncharacterized [66]. As previously discussed, PNAG synthesis is driven by c-di-GMP, whose homeostasis is finely regulated by diguanylate cyclases and phosphodiesterases [67]. In agreement with the increased PNAG levels, the Δ*arcZ* strain exhibited elevated c-di-GMP levels, similar to the Δ*hfq* strain, suggesting that ArcZ plays a modulatory role in c-di-GMP metabolism and, consequently, in biofilm matrix production.

Interestingly, among the predicted targets of ArcZ appears RovM, which is an important transcriptional regulator within prokaryotic organisms, particularly in the genus *Yersinia*, where it functions modulating diverse physiological and virulence-associated processes. As a member of the LysR-like regulator family, RovM is involved in the expression regulation of virulence determinants such as invasin and flagellar genes, and in biofilm development, responding to environmental stimuli such as nutrient availability and temperature variations [68,69]. A similar regulatory framework could allow *Y. ruckeri* to adapt to variable environmental conditions while optimizing their survival strategies and infective potential. In addition, the negative regulatory effect of ArcZ on *rovM* expression is particularly noteworthy, as it suggests the presence of a coherent feedforward loop involving RovM, FlhDC, and PdeH. This regulatory motif aligns with the phenotypes observed in the Δ*arcZ* strain. In the absence of ArcZ, *rovM* is upregulated, which may lead to the increased expression of the flagellar master regulator *flhDC* since RovM is known to activate *flhDC* expression in *Y. pseudotuberculosis* [32]. This upregulation likely contributes to the hypermotility phenotype observed in the Δ*arcZ* strain. Furthermore, the subsequent downregulation of *pdeH* expression, possibly due to repression by FlhDC, as reported in *E. coli* [33], results in elevated intracellular c-di-GMP levels and the formation of denser, polysaccharide-rich biofilms. In *E. coli*, PdeH is recognized as the primary phosphodiesterase responsible for degrading cellular c-di-GMP, thereby modulating c-di-GMP-dependent pathways [70]. As a globally acting enzyme, PdeH counterbalances the activity of multiple diguanylate cyclases, a function that is critical for the bacterial transition between motile and sessile states.

Regarding the role of *Yersinia* sRNAs in regulating genes involved in c-di-GMP metabolism and biofilm formation, previous studies in *Y. pestis* have demonstrated that the chromosomally encoded sRNA HmsB stimulates the synthesis of c-di-GMP and PNAG by upregulating the diguanylate cyclase genes *hmsT* and *hmsD*, as well as the *hmsHFRS* operon responsible for PNAG synthesis and export [71]. Moreover, HmsB represses the expression of the phosphodiesterase gene *hmsP* [71]. In contrast, the plasmid-derived sRNA HmsA not only activates the same targets as HmsB but also upregulates *hmsB*, *rovM*, and *hmsD*, while repressing the transcription of the biofilm repressor RovA [72]. Notably, in the *Y. ruckeri* strain CD2 used in this study, the genes encoding these sRNAs were not detected.

In *Erwinia amylovora*, ArcZ modulates both motility and biofilm formation by directly repressing *flhDC* expression and indirectly influencing transcription through the repression of the transcriptional repressor Lrp [73]. Similarly, in *E. coli*, ArcZ directly inhibits *flhDC* translation [48] and indirectly promotes the synthesis of biofilm matrix components, such as curli fibers and cellulose, by activating RpoS translation [61]. In *S. enterica,* sv. Typhimurium biofilm formation is primarily governed by CsgD, a transcriptional activator of curli fibers and cellulose [61], and ArcZ further regulates surface adhesion by repressing the synthesis of type 1 fimbriae [62].

The role of RybB in modulating biofilm formation has been documented in *E. coli* under envelope stress induced by green tea polyphenols, which inhibit biofilm development [59]. In these conditions, the transcriptional factor RpoE activates RybB expression, and RybB, in turn, represses CsgD, thereby suppressing the synthesis of curli fibers and cellulose [59]. An autoregulatory loop exists in *E. coli*, wherein RpoE both regulates and is regulated by RybB [74]. Moreover, RybB, together with Hfq, has been shown to downregulate outer membrane proteins (OMPs) such as OmpC and OmpW under envelope stress in both *E. coli* and *S. enterica* [20,74,75,76,77].

In our study, RpoE was predicted as a direct target of RybB, and its expression was significantly upregulated in the absence of RybB, indicating that RybB normally acts as a repressor of RpoE. This observation raises the possibility of a feedforward loop involving RybB, RpoE, and additional factors that directly influence biofilm maturation and matrix polysaccharide synthesis, similar to mechanisms described in *E. coli* [59]. RpoE is a sigma factor widely characterized at the molecular level regarding its response to cell envelope stress. In a broad context, any form of environmental perturbation that leads to the accumulation of misfolded proteins within the periplasm, including but not limited to oxidative stress, osmotic pressure, variations in pH, or exposure to antimicrobial compounds, results in the activation of the RpoE signaling pathway [78]. Furthermore, the activity of RpoE is stimulated upon entry into the stationary phase [79] and plays a significant role in biofilm formation as well as conferring resistance to antimicrobial agents [80]. Fundamentally, RpoE functions by integrating diverse signals, all of which drive to malfunctions in the biogenesis of the outer membrane, thereby initiating a protective response that activates cellular damage repair pathways. This adaptive mechanism encompasses the transcription of genes associated with the biogenesis, transport, and/or assembly of lipopolysaccharides, phospholipids, OMPs, and various proteases and chaperones that are essential for maintaining or repairing outer membrane integrity [20]. Thus, the extent or impact that RybB can have on regulating RpoE expression under stress conditions leading to biofilm formation is potentially very relevant.

Based in our findings, it is plausible that RybB modulates the temporal expression of RpoE and OMPs (e.g., OmpW and OmpC) during specific stages of *Y. ruckeri* biofilm development or under particular environmental conditions. OMP expression plays a critical role in the early stages of biofilm formation by mediating cell-to-cell and cell-to-surface adhesion. For example, in *Vibrio cholerae*, the outer membrane protein OmpU contributes to the biofilm matrix by modulating initial adhesion and polysaccharide production [81]. Conversely, under stress conditions such as antimicrobial exposure, the repression of OMPs may be necessary, as their presence can facilitate antibiotic entry into bacterial cells [82]. Thus, sRNAs like RybB and ArcZ, along with other regulatory RNAs, likely function as sophisticated modulators, balancing gene expression networks to fine-tune biofilm development and adaptation to varying environmental stimuli.

The increased biofilm formation by the absence of Hfq or sRNAs is a phenomenon observed across different bacterial species and can be attributed to the multifaceted role of the Hfq and sRNAs as post-transcriptional regulators of many targets. Both Hfq and sRNAs are known to interact with sRNAs and mRNAs [83], [84], including other global regulators, thus influencing various cellular processes. The absence of these regulators disrupts diverse regulatory pathways, triggering in turn stress and/or compensatory responses that ultimately can enhance biofilm formation. This does not necessarily imply that these biofilms and the bacteria embedded therein are in optimal homeostatic conditions to cope with a multifactorial environment. It is plausible that these biofilms exhibit metabolic imbalances that make them more susceptible to multiple external factors. Further studies are needed to characterize the susceptibility of these mutant biofilms

Comparing our findings with those from other bacterial systems underscores the complex regulatory networks governing biofilm formation. While some sRNAs and regulatory factors are conserved across species, more specific regulators also emerge. Consequently, the regulatory cascades and network motifs can vary between genera and even among species within the same genus, leading to diverse biofilm outcomes. Furthermore, the temporal dynamics of gene expression during biofilm development add another layer of complexity, as individual regulators may exhibit different expression patterns in response to fluctuating signals and stimuli. Ultimately, the varied biofilm phenotypes observed across bacteria reflect evolutionary adaptations to the distinct ecological niches these pathogens inhabit and the hosts they colonize.

### 3.4. Future Perspectives and Challenges 

Despite its critical role in pathogenicity, several components of biofilm formation in *Y. ruckeri* remain uncharacterized. For example, the biofilm adhesin polymer cluster and the enzymes involved in c-di-GMP metabolism in the *Y. ruckeri* genome have yet to be fully elucidated. This gap limits our understanding of how these factors interact to regulate biofilm development. Moreover, the broader molecular mechanisms and factors underlying biofilm formation in this pathogen remain largely unknown. In particular, the sRNA-mediated regulation of specific targets by both conserved and novel sRNAs identified in our study needs further investigation to clarify their roles within the biofilm regulatory network.

Overall, our results provide compelling evidence that the Hfq chaperone and its associated sRNAs are key regulators of biofilm formation in *Y. ruckeri*. Although the genetic and mechanistic bases of biofilm formation are well characterized in other Enterobacteriaceae, it is essential to consider the unique evolutionary and ecological contexts of *Y. ruckeri*. Biofilm formation not only represents a vital survival strategy but also enhances virulence and transmission in this pathogen. The complex, multilayered regulatory networks controlling biofilm development underscore the remarkable adaptability of bacteria to diverse environments and hosts. A deeper understanding of these mechanisms may reveal novel targets for disrupting biofilm formation and controlling infections caused by *Y. ruckeri* and related species, for instance, to study possible regulatory interactions between sRNAs and quorum-sensing systems to develop specific quorum-quenching strategies.

## 4. Materials and Methods

### 4.1. Bacterial Strains and Culture Conditions

*E. coli* cultures were grown in Luria–Bertani (LB) (US BIO, Salem, MA, USA) medium supplemented with ampicillin (100 µg/mL) (Winkler, Stgo, Chile) or kanamycin (50 µg/mL) (Sigma-Aldrich, St. Louis, MO, USA), as required, incubated at 37 °C with constant shaking until reaching an OD_600_ of 1.1. For the LB agar medium, LB was supplemented with 2% (*w*/*v*) agar-agar (Winkler, Santiago, Chile) and antibiotics, as required. For *Y. ruckeri* cultures, Trypticase Soya (TSB) medium (BD Difco™, New Jersey, MD, USA), containing 17 g/L casein pancreatic digest, 3 g/L soybean pancreatic digest, 5 g/L NaCl, 2.5 g/L K_2_HPO_4_, and 2.5 g/L dextrose, was used. For solid and semi-solid Trypticase Soy Agar (TSA) media, TSB medium was supplemented with 1.2%, 0.5%, or 0.3% (*w*/*v*) agar-agar (Winkler, Stgo, Chile). *Y. ruckeri* cultures were incubated at 26 °C with shaking for until reaching an OD_600_ of 1.1. For biofilm formation assays, cultures at OD_600_ of 1.1 were diluted to an OD_600_ of 0.5, in order to normalize the initial cell number to 4 × 10^8^ CFU. These assays were carried out without shaking, at 22 °C. The strains used in this study are indicated in Supplementary Table S2.

To obtain mutant strains deficient in the *rprA*, *arcZ*, and *rybB* genes, the unmarked allelic exchange recombination technique was performed as previously described for *hfq* mutant strain [85]. First, a sequence of approximately 500 nt upstream and downstream of the gene to be mutated (referred as “arm 1” and “arm 2”), were amplified by PCR using the genomic DNA of the *Y. ruckeri* CD2 strain as the template. By a second PCR was obtained a linear product of approximately 1000 bp, using “arm 1” and “arm 2” as templates, which contained a 10 bp for complementarity at the 3’ and 5′ ends, respectively. This linear product was digested with EcoRI (Thermo Fisher, Waltham, MA, USA) and SacI (Thermo Fisher, Waltham, MA, USA), for 3 h at 37 °C. In addition, the pFOK plasmid [86] (pFOK was a gift from Dirk Bumann [Addgene plasmid #166651, http://n2t.net/addgene:166651; accessed on 14 March 2025, RRID:Addgene_166651]) was digested with EcoRI and SacI. The products obtained from digestions were purified using the Gel Extraction Kit (Omega BioTek, Norcross, GA, USA) according to the manufacturer’s instructions. The digested PCR products were ligated into linearized pFOK vector at a 3:1 ratio using the T4 DNA Ligase enzyme (Promega, Madison, WI, USA), according to the manufacturer’s instructions. The recombinant vectors were transformed into competent *E. coli* DH5-α cells and grown in LB medium at 37 °C with shaking for 1 h. For mutant strain selection, strains grown on TSA plates supplemented with kanamycin (100 µg/mL) were subsequently seeded on solid medium without salt, containing tryptone (17 g/L) (UsBiological, Salem, MA, USA), and kanamycin (100 µg/mL), soytone (3 g/L) (UsBiological, Salem, MA, USA), anhydrous glucose 2.5 g/L (UsBiological, Salem, MA, USA), dibasic potassium phosphate (Merck, Darmstadt, Germany), sucrose 20% (*w*/*v*) (Santa Cruz, Dallas, TX, USA), and agar-agar 1.2% (*w*/*v*) (BD Difco™, New Jersey, MD, USA). In addition, plates were supplemented with anhydrotetracycline (10 µg/mL) (Santa Cruz, Dallas, TX, USA). The clones obtained from *E. coli* DH5- α were verified by PCR using specific oligos for pFOK (Supplementary Table S3).

For conjugation into *Y. ruckeri*, the recombinant vectors were purified and transformed into the *E. coli* S-17λ-pir donor strain. The donor strain was grown in LB medium supplemented with streptomycin (50 µg/mL) (PhytoTech Labs, Lenexa KS, USA) and kanamycin (50 µg/mL) to an OD_600_ of 0.6 at 37 °C with shaking. The recipient strain *Y. ruckeri* was grown in TSB medium to an OD_600_ of 0.6 at 26 °C with shaking. Mixtures were made at different ratios of donor vs. recipient strain (1:1; 3:1 and 5:1). Cells were centrifuged at 5400× *g* and washed twice with sterile PBS (Thermo Fisher, Waltham, MA, USA). Then, they were suspended in 100 µL of sterile PBS and spotted onto a TSA plate and incubated at 30 °C for 24 h. After incubation time, the cell mass was recovered by the addition of 500 µL sterile PBS. To select cells that successfully incorporated the vector into their chromosome (first recombination event), the recovered volume was plated on TSA plates supplemented with kanamycin (100 µg/mL) and ampicillin (300 µg/mL). Ampicillin was used to eliminate the donor strain, since *Y. ruckeri* CD2 strain is resistant to this antibiotic. Plates were incubated overnight at 26 °C, and the resulting colonies were grown in 1 mL of TSB medium supplemented with kanamycin (100 µg/mL) in 24-well plates (Corning Inc., Corning, NY, USA) until reaching an OD_600_ of 0.4. At this point, an aliquot of 100 µL was taken and seeded onto a TSA plate supplemented with 10% (*w*/*v*) sucrose and anhydrotetracycline (10 µg/mL) (AppliChem, Darmstadt, Germany) to allow for the second recombination event and subsequent negative selection. Finally, the negative selection of colonies obtained by kanamycin sensitivity in liquid medium was performed. Those colonies that failed to grow in the TSB medium supplemented with kanamycin and did grow in the TSA medium supplemented with 10% sucrose (Merk, Darmstadt, Germany) were verified by PCR.

### 4.2. Motility Assays

For swimming assays, strains were cultured in TSB medium until reaching an OD_600_ of 1.1. Then, 1 µL was inoculated onto 0.3% (*w*/*v*) TSA plates and incubated at 26 °C for 24 and 48 h. For swarming assays, strains were grown in TSB medium until reaching an OD_600_ of 1.1 to then inoculate 1 μL onto the surface of 0.5% (*w*/*v*) TSA plates. Plates were incubated at 26 °C for 24 and 48 h. For both experiments, images were captured with a G:BOX Chemi XRQ photodocumenter (Syngene, Cambridge, UK).

### 4.3. Biofilm Formation Assay

For all biofilm formation assays, *Y. ruckeri* strains were grown in TSB medium at 26 °C with shaking until reaching an OD_600_ of 1.1. Then, the cultures were diluted to an OD_600_ of 0.5 in fresh TSB medium, and 1 mL aliquots of these dilutions were deposited in 24-well plates, containing a 12 mm silicate covers (Marienfeld, Lauda-Königshofen, Germany) on the bottom. Plates were incubated at 22 °C without shaking for 4, 6, 12, 24, and 48 h. After each incubation interval, covers were recovered, washed twice with sterile PBS by immersion, and treated as indicated for each analysis.

### 4.4. Crystal Violet Staining Assay

After each incubation time for biofilm formation, the covers were washed, air-dried for 10 min, and then fixed with 200 µL of 100% (*v*/*v*) methanol (Winkler, Santiago, Chile). The fixed biofilms were transferred to a new 24-well plate, containing 1 mL of 1% (*v*/*v*) Crystal Violet solution (Winkler, Santiago, Chile), and incubated for 20 min at room temperature. Then, the covers were washed twice with sterile PBS by gentle immersion and air-dried. Finally, the biofilms were solubilized with 500 µL of a 33% (*v*/*v*) acetic acid solution (Winkler, Santiago, Chile).

### 4.5. Macrocolony Formation in Congo Red Plates

Congo red plates were prepared by supplementing TSA with 0.08% (*v*/*v*) sterile Congo red (Winkler, Santiago, Chile). *Y. ruckeri* strains were cultured in TSB medium until reaching an OD_600_ of 1.1. Then, 1 µL was inoculated onto Congo red plates and incubated at 22 °C for 72 h to capture images of the macrocolonies.

### 4.6. Analysis of Biofilms by Confocal Microscopy

For confocal microscopy analysis, *Y. ruckeri* strains were previously transformed with the pDiGc plasmid (pDiGc was a gift from Sophie Helaine & David Holden [Addgene plasmid #59322, http://n2t.net/addgene:59322; accessed on 14 March 2025, RRID:Addgene_59322]) to visualize the cells. After each incubation time of biofilm formation, the silicate covers were recovered, washed twice with sterile PBS, and fixed with 4% (*v*/*v*) paraformaldehyde (Electron Microscopy Science, Hatfield, PA, USA) for 20 min at room temperature. Once fixed, covers were deposited on a slide with 10 µL of Fluoromount-G mounting medium (Thermo Fisher, Waltham, MA, USA), air-dried for 10 min in a dark room, and stored at 4 °C. For the analysis of extracellular polysaccharides, the samples fixed with 4% (*v*/*v*) paraformaldehyde were stained with 100 µL of Wheat Germ Agglutinin (WGA) conjugated with AlexaFluor 647 (Thermo Fisher, Waltham, MA, USA) for 20 min at room temperature in dark. Then, the covers were washed twice with sterile PBS and deposited on slides with 10 µL of Fluoromount-G mounting medium. The samples were analyzed on a Leica TCS SP8 confocal optical microscope (Leica Microsystems GmbH, Wetzlar, Germany).

### 4.7. Biofilm Architecture Analysis by Scanning Electron Microscopy

After each incubation time of biofilms, the covers were recovered, washed twice with sterile PBS, and fixed with 500 µL of 2.5% (*v*/*v*) glutaraldehyde (Electron Microscopy Science, Hatfield, PA, USA). The samples were processed by critical point drying and gold shading. The analyses were performed with a Hitachi TM3000 scanning electron microscope (Hitachi High-Tech, Tokyo, Japan).

### 4.8. Quantification of Cyclic Diguanosine Monophosphate (c-di-GMP)

Intracellular levels of c-di-GMP were quantified using a Cyclic-di-GMP Assay Kit (Lucerna Technologies, Brooklyn, NY, USA) and following the manufacturer′s guidelines. Briefly, biofilm samples were washed twice with sterile PBS and deposited in 24-well plates containing 500 µL of sterile PBS, and biofilm cells were recovered by applying three pulses of low-frequency sonication (Elma, Singen, Germany), for 30 s each one. Then, 70 µL of each sample of the recovered cells was supplemented with a serially diluted c-di-GMP standard and the assay reactions set up according to the manufacturer’s instructions. Subsequently, the mixture was incubated at room temperature for 30 min in dark. The fluorescence intensity was determined in a Synergy H1 Microplate Reader fluorometer (Biotek, San Diego, CA, USA) at λ 469/501 nm of ex/em. The concentration of c-di-GMP was calculated according to the standard calibration curve, and the colony forming units were determined from the biofilm samples to normalize the concentration of c-di-GMP.

### 4.9. Genome Assembly and Annotation

Genomic DNA was sequenced using the DNBSEQ-G400 platform (MGI Tech Co., Ltd., Shenzhen, China), generating 150 bp paired-end reads. Genome assembly was performed with SPAdes v. 3.15.3 [87], with k-mer sizes of 21, 33, 55, 77, 99, and 127 and using a pre-assembly with the flag –untrusted-contigs. A polishing step was conducted using Pilon v1.24 [88]. Sequencing reads were aligned to the draft-assembly with BWA, and the resulting alignments were processed with SAMtools to generate sorted and indexed BAM files. These files were then used by Pilon to correct potential sequencing errors and improve overall quality. The quality of the polished genome was further assessed using QUAST (https://github.com/ablab/quast (accessed on 14 March 2025)) to confirm assembly metrics and ensure consistency. Contigs smaller than 200 bp were filtered out using SeqKit v.2.10.0 [89]. Raw sequencing data were deposited in the Sequence Reads Archive under accession number PRJNA1162115, and the Quality Control (QC) report of raw and trimmed files is included in Supplementary Table S4. Gene calling and annotation were performed using Bakta v1.10.4 [90].

### 4.10. RNA-Seq and sRNAome Analyses

Fifteen samples of *Yersinia ruckeri* CD2 were collected in triplicate at 0 h, 4 h, 6 h, 12 h, and 24 h under biofilm-forming conditions. Total RNA extraction was performed by GeneJET™ RNA Purification Kit (Thermo Scientific™, Waltham, MA, USA) following manufacturer’s instructions, and trace amounts of DNA present in the samples were digested with DNase I (Thermo Scientific™, Waltham, MA, USA) for 90 min at 37 °C in a T960 thermal cycler (Heal Force, Shanghai, China). Total DNA digestion was corroborated by PCR amplification, with housekeeping gyrA gene oligos. RNA libraries were prepared using the Illumina TruSeq Small RNA Kit and sequenced on an Illumina MiSeq platform (151 bp, single-end). Raw data are available in the Sequence Read Archive (SRA) under Bioproject PRJNA1162115. Reads were trimmed and filtered with Cutadapt v5.0 [91] and Fastp v0.24.0 [92], and then aligned to the *Y. ruckeri* CD2 reference genome using BWA v0.7.19. Gene counts were obtained with featureCounts using a non-coding RNA annotation file generated with Bakta v1.11.0 (DB v6.0.0). A differential expression analysis was performed in R with package DESeq2 v1.46.0 [93], applying thresholds of adjusted *p*-value ≤ 0.05 and |log₂ fold change| ≥ 1.5. Expression levels were normalized and scaled as Z-scores. All data visualization and differential expression plots were generated using R (version 4.4.1) with the packages ‘ggplot2’ v 3.5.2, ‘EnhancedVolcano’ (release 23 July 2021), and ‘ComplexHeatmap’ (https://github.com/jokergoo/ComplexHeatmap (accessed on 14 March 2025)).

### 4.11. RT-qPCR Analysis

Gene expression quantification analysis was performed from the biofilm cells of *Y. ruckeri* strains diluted in sterile TSB at an OD_600_ of 0.5. Total RNA extraction was performed by the TRIzol reagent (Invitrogen™, Waltham, MA, USA), following the manufacturer’s instructions, and trace amounts of DNA present in the samples were digested with DNase I (Thermo Scientific™, Waltham, MA, USA) for 90 min at 37 °C in a T960 thermal cycler (Heal Force, Shanghai, China). Total DNA digestion was corroborated by PCR amplification, with housekeeping gyrA gene oligos. The cDNA synthesis was performed with 1 µg of each RNA sample using specific oligos of all the genes to be analyzed, at a concentration of 10 mM. The reaction was carried out with the M-MLV Reverse Transcriptase enzyme (Promega, Madison, WI, USA), following the manufacturer’s instructions. Finally, gene expression levels were determined using the KAPA SYBR^®^ FAST qPCR kit (KAPA Biosystems, Wilmington, MA, USA), according to the manufacturer’s instructions, in a AriaMx Real-Time PCR system Mx3000P (Agilent Technologies, Santa Clara, CA, USA). The housekeeping gene *gyrA* was used for the normalization of gene expression.

### 4.12. Statistics

The data were statistically analyzed with a Student’s *t*-test. Values of *p* < 0.05 were considered statistically significant.

## Data Availability

The raw sequencing data were deposited in the NCBI Sequence Read Archive (SRA) under accession number PRJNA1162115.

## Supplementary Materials

The supporting information can be downloaded at: https://www.mdpi.com/article/10.3390/ijms26104733/s1.
