# Supplementary material for: The RNA Chaperone Hfq and Small Non-Coding RNAs Modulate the Biofilm Formation of the Fish Pathogen Yersinia ruckeri
Source: Int J Mol Sci. 2025 May 15;26(10):4733. doi: 10.3390/ijms26104733 (PMC12112432; doi:10.3390/ijms26104733)
Supplement: Supplementary file 1 [file ijms-26-04733-s001.zip › Supplementary Figures MS ID 3621475.pdf]

Supplementary figures. Barros *et al.*, 2025, IJMS.

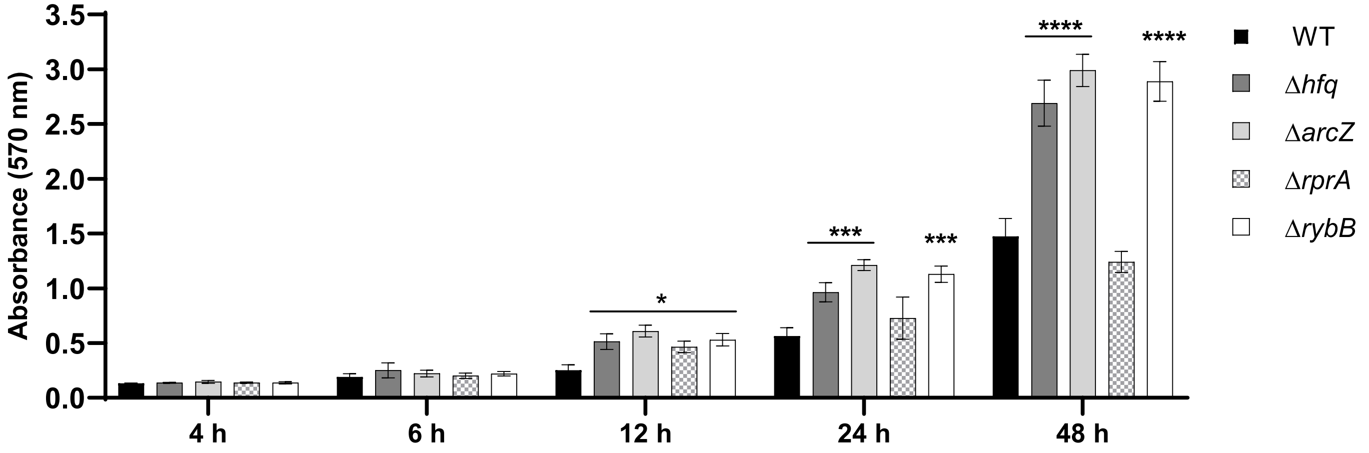

Figure Supplementary S1. Crystal Violet biofilm assays of *Y. ruckeri* strains. Wild-type (WT), Δ*hfq*, and ΔsRNA strains were grown on silicate covers without shaking at 22 °C for the indicated durations (4, 6, 12, 24, and 48 h). The covers were then stained with 1% (v/v) Crystal Violet to visualize biofilm formation. Asterisks represent statistically significant differences with respect to the WT (****$p < 0.0001$; ***$p < 0.001$, *$p < 0.1$). Data represent the means ± standard deviations (n= 3).

A)

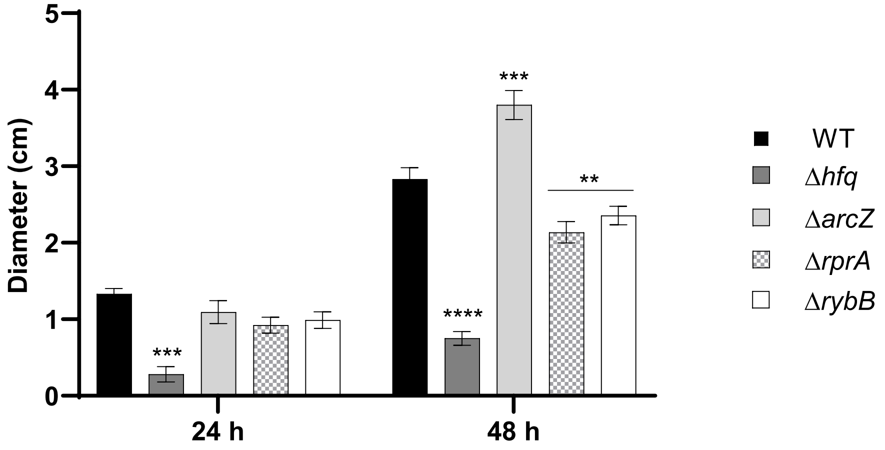

B)

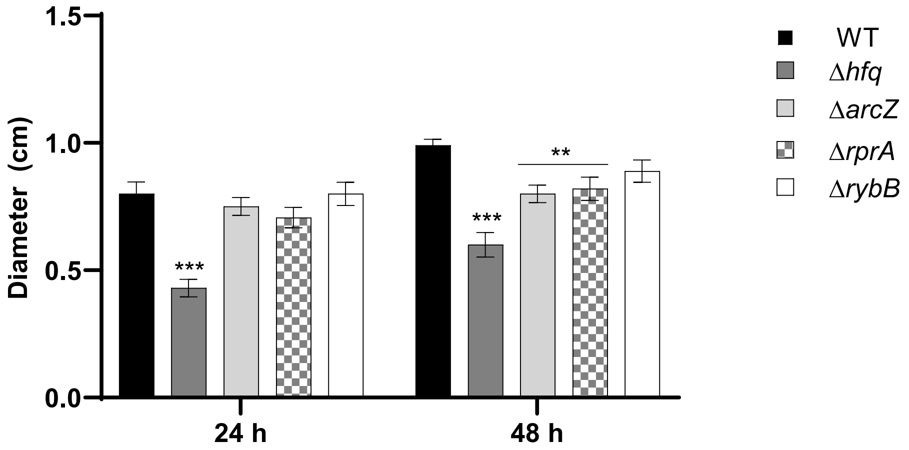

Figure S2. Motility assays of *Y. ruckeri* strains. A) Late-log phase cultures (1 μL) of wild-type (WT), Δ*hfq*, and ΔsRNA strains were spotted onto TSA agar plates containing 0.3% agar. Swimming motility was evaluated after 24 and 48 h of incubation by determination of motility halo diameter. Asterisks represent statistically significant differences with respect to the WT (****$p < 0.0001$; ***$p < 0.001$, **$p < 0.01$). Data represent the means ± standard deviations (n= 5). **B)** Late-log phase cultures (1 μL) of Wild-type (WT), Δ*hfq*, and ΔsRNA strains were spotted onto TSA agar plates containing 0.5% agar. Swarming motility was evaluated after 24 and 48 h of incubation by determination of motility halo diameter. Asterisks represent statistically significant differences with respect to the WT (***$p < 0.001$, **$p < 0.01$). Data represent the means ± standard deviations (n= 5).
